# E7-Conjugated Bio-Inspired Microspheres as a Biological Barrier for Guided Tissue Regeneration

**DOI:** 10.1021/acsami.3c12213

**Published:** 2023-12-08

**Authors:** Zhiai Hu, Xin Rong, Xiaohua Liu

**Affiliations:** † Department of Biomedical Sciences, Texas A&M University School of Dentistry, Dallas, Texas 75246, United States; ‡ Chemical and Biomedical Engineering Department, University of Missouri, Columbia, Missouri 65211, United States

**Keywords:** guided tissue regeneration, periodontal, alveolar bone, regeneration, E7 peptide

## Abstract

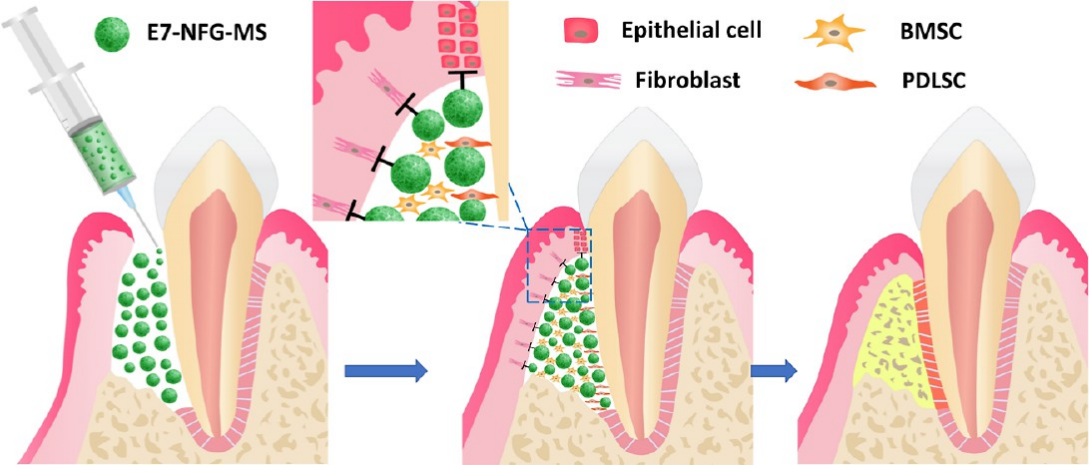

Guided tissue regeneration (GTR), which is based on creating a physical barrier to prevent the downgrowth of epithelial and connective tissues into the defect site, has been widely used in clinical practice for periodontal regeneration for many years. However, its outcomes remain variable due to highly specific indications, the demand for proficient surgical skills, and frequent occurrence of complications. In this study, we developed a new GTR biomaterial that acts as a biological barrier for epithelial cells and fibroblasts while also serving as a scaffold for bone marrow-derived mesenchymal stem cells (BMSCs) and periodontal ligament stem cells (PDLSCs). This innovative GTR biomaterial is bioinspired injectable microspheres that are self-assembled from nanofibers, and their surfaces are conjugated with E7, a short peptide that selectively promotes BMSC and PDLSC adhesion but inhibits the attachment and spreading of epithelial cells and gingival fibroblasts. The selective affinity afforded by E7 on the surfaces of the nanofibrous microspheres facilitated the colonization of BMSCs in the periodontal defect, thereby substantially improving functional periodontal regeneration, as evidenced by enhanced new bone formation, reduced root exposure, and diminished attachment loss. The remarkable superiority of the bioinspired microspheres over conventional GTR materials in promoting periodontal regeneration underscores the potential of this innovative approach to enhance the efficacy of functional periodontal tissue regeneration.

## 1 Introduction

The periodontium, a functional unit that anchors teeth into the jaws, is composed of the gingiva, cementum, periodontal ligament (PDL), and alveolar bone. Periodontitis is an inflammatory condition of the periodontium, and it progressively destroys the tissues surrounding the tooth, ultimately leading to tooth mobility and tooth loss.^1^ Severe periodontitis is the sixth most prevalent disease worldwide, affecting more than one billion people worldwide.^2^ Moreover, the prevalence of periodontitis is expected to increase as the population ages, presenting a significant public health challenge.^3^ Periodontitis is commonly treated through conventional therapies (e.g., scaling and root planning, antibiotics, and flap surgery) to remove local pathogenic factors like plaque and tartar, eliminate inflammation, and ultimately alleviate or halt the progression of the disease.^4^ Although these therapies can be effective, they do not address the problem of periodontal tissue loss, and the function of the periodontium remains compromised, which undermines the long-term efficacy of the treatments.

**Figure 1:**
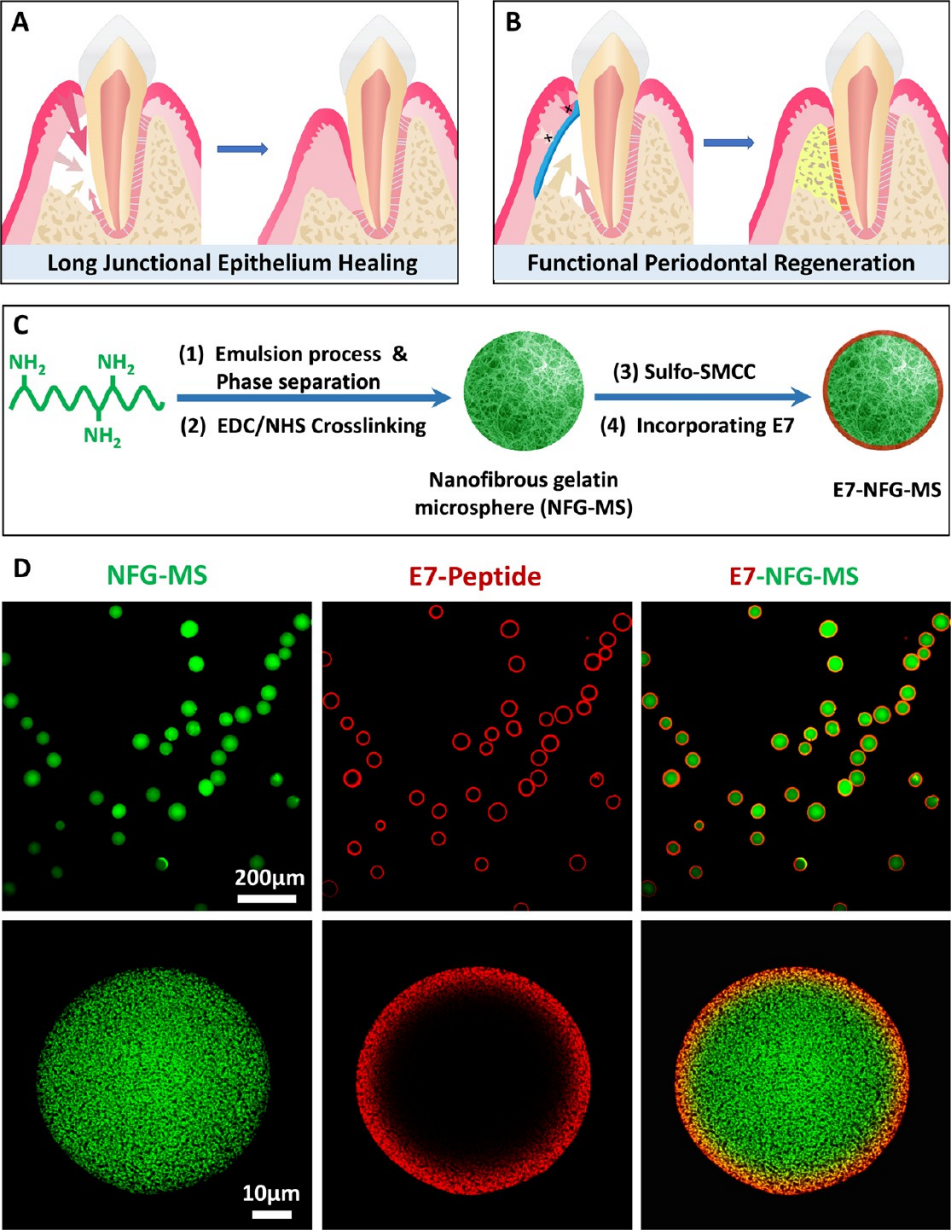
(A,B) Schematic diagram of long junctional epithelium healing and functional periodontal regeneration of periodontal defect. (C) Schematic diagram of the synthesis of NFG-MS and E7-NFG-MS. (D) Cross-section confocal images of E7-NFG-MS.

Periodontal tissue regeneration has been proposed to reconstruct the lost periodontal tissues and restore their functions.^5,6^ There are at least four types of cells that are involved in periodontal regeneration, including gingival epithelial cells (GEFs), gingival fibroblasts (GFs), PDL stem cells (PDLSCs), and bone marrow-derived mesenchymal stem cells (BMSCs).^7^ During the natural healing process of periodontal defects, GEFs and GFs from the flap initially occupy the denuded root surface and the defect area, leading to the formation of a long junctional epithelium or fibrous connective tissue (Figure 1A).^8−10^ This process impedes the migration of PDLSCs and BMSCs to the defect area and prevents the formation of a functional cementum-PDL-alveolar bone complex, resulting in the failure to rebuild the lost PDL and alveolar bone.^8^

Guided tissue regeneration (GTR) is utilized to reverse the unfavorable cell-space-occupying sequence and is currently the most used technique for periodontal tissue regeneration in clinical practice. GTR involves applying a barrier membrane to cover the periodontal defect, creating a space for the inward migration of PDLSCs and BMSCs, while preventing the ingrowth of undesired GEFs and GFs (Figure 1B).^11^ The GTR barrier membranes can be divided into nonresorbable and resorbable membranes.^12^ While nonabsorbable membranes have sufficient stiffness to maintain space stability, a secondary surgery is necessary to retrieve them, which increases the risk of site morbidity. In addition, nonabsorbable GTR membranes often have high premature exposure rates, leading to significant increases in infection, contamination, and impaired bone augmentation.^12^ Absorbable membranes can avoid the need for a second surgery, but their poor mechanical properties make them prone to collapse and displacement. Recently, electrospinning and melt electrowriting (MEW) technologies were developed to fabricate biomimetic GTR membranes with tunable physical and biological properties.^13−16^ Despite considerable efforts to overcome these limitations, the GTR membranes for periodontal regeneration are successful only in some selective cases, such as narrow intrabony defects and class II mandibular furcation defects.^17^ Therefore, there is an unmet need to develop new strategies for periodontal regeneration.

To achieve successful periodontal regeneration, it is essential to repopulate the defect region with PDLSCs and BMSCs, rather than GEFs and GFs.^7^ Recently, a peptide known as EPLQLKM (E7) was identified to possess a specific affinity for MSCs.^18,19^ It was reported that collagen substrates modified with the E7 peptide selectively captured and adhered to MSCs while preventing fibroblasts and inflammatory cells from attachment.^20^ However, it remains unclear whether the E7 peptide can selectively facilitate the adhesion of PDLSCs and BMSCs while simultaneously preventing the ingrowth of GEFs and GFs when it is used for periodontal regeneration.

In this study, a type of distinct injectable microsphere, inspired by natural bone extracellular matrices (ECM), was fabricated as a scaffolding biomaterial with a specific affinity for BMSCs and PDLSCs. The microspheres were self-assembled from gelatin nanofibers, simulated both the chemical composition and the physical architecture of natural bone ECM, and exhibited excellent biocompatibility and osteoconductivity.^21,22^ As an injectable scaffold, the microspheres can easily fill the irregular periodontal defects in a minimally invasive manner and provide sufficient macropores and micropores for nutrient exchange to facilitate new tissue regeneration.^23^ More importantly, the E7 peptide was immobilized onto the surface of the nanofibrous microspheres to selectively capture the BMSCs and PDLSCs. We hypothesized that the E7-conjugated bioinspired microspheres would serve not only as a scaffold to promote the adhesion and growth of BMSCs and PDLSCs, but also as a biological barrier to inhibit the attachment of GEFs and GFs, therefore leading to a significant enhancement of periodontal regeneration.

## 2 Materials and Methods

### 2.1 Fabrication of Nanofibrous Gelatin Microspheres

Nanofibrous gelatin microspheres (NFG-MSs) were synthesized using a combination of the water/oil emulsification technique and the thermally induced phase separation process, as previously reported.^22,24,25^ Briefly, gelatin was dissolved in a 50% ethanol solvent at 50 °C and added to mineral oil under rigorous mechanical agitation to form a water/oil emulsion. The resulting mixture was poured into an isopropanol/1,4-dioxane/ethanol solvent mixture at −20 °C to induce phase separation and solvent exchange. The obtained NFG-MS were cross-linked with 1-ethyl-3-(3-(dimethylamino)propyl) carbodiimide (EDC) and *N*-hydroxy succinimide (NHS) at 4 °C, followed by incubation with glycine solution to neutralize the unreacted cross-linker. The NFG-MS were then washed with distilled water, sieved to obtain different sizes, and finally freeze-dried for subsequent use. The protocols used for the characterizations of the NFG-MS, including fiber diameters, morphology, porosity, apparent density, degradation, and swelling rate, were described in our previous publications.^25−28^ It is worth noting that in order to accelerate the degradation process in vitro, a relatively high concentration of collagenase (5 units/mL) was added to the degradation medium. Three samples were obtained at each time point, and the experiment was repeated three times.

### 2.2 Conjugation of E7 Peptide onto NFG-MS

E7-peptide (EPLQLKM) was synthesized via solid-phase peptide synthesis using Fmoc Chemistry (Scilight-Peptide Inc., Beijing, China). Additional cysteine was introduced at the carboxyl (C) terminus of E7 to facilitate scaffold conjugation and rhodamine labeling. The peptide was dissolved in dimethyl sulfoxide (DMSO) to achieve a stock concentration of 2 mg/mL. The conjugation of E7 peptide onto NFG-MS was performed as previously described.^18^ Briefly, 5 mg of NFG-MS was first washed three times with 0.1 M phosphate buffered saline (PBS) containing 0.15 M NaCl (Thermo Fisher Scientific). Next, the NFG-MS was immersed in 1 mL of sulfosuccinimidyl-4-(*N*-maleimidomethyl) cyclohexane-1-carboxylate (sulfo-SMCC, Thermo Fisher Scientific) solution (2 mg/mL) for 1 h at room temperature with gentle stirring. After the NFG-MS was washed three times with PBS containing 0.1 M EDTA at pH 7.0, the microspheres were incubated in 1 mL of E7 peptide solution (0.2 mg/mL) at room temperature for 1 h. The E7-conjugated NFG-MS were then washed three times with distilled water, lyophilized, and stored at 4 °C. To visualize the distribution of the E7 peptide on the NFG-MS, the gelatin was labeled with fluorescein-5-isothiocyanate (FITC) and the E7 peptide was labeled with rhodamine. After the conjugation, E7-NFG-MS was observed with a confocal laser scanning microscope.

### 2.3 Isolation of GECs, GFs, PDLSCs, and BMSCs

Male Sprague–Dawley (SD) rats aged 4 weeks were used to isolate GECs, GFs, PDLSCs, and BMSCs. The isolation of GEC and GF was performed according to the method previously described with minor modifications.^29,30^ Specifically, the gingival tissue was disinfected twice with povidone-iodine and washed with PBS before being incubated in Dispase II (Sigma) to separate the epithelial and connective tissue. The epithelial tissue was cut into small pieces and cultured in DMEM with 10% fetal bovine serum (FBS) to promote the outward migration of GECs. GFs were isolated from the connective tissue by incubation in collagenase (Sigma) and cultured in α-MEM with 10% FBS. For PDLSC isolation, the PDL tissue from rat molars was enzymatically digested with collagenase and trypsin before being filtered and cultured in α-MEM supplemented with 10% FBS.^31^ BMSCs were obtained by flushing the marrow cavity of rat femurs and tibias with α-MEM, filtering the cells, and culturing them in α-MEM with 10% FBS after red blood cell lysis.^21^ The cells at passages 2–4 were used in this study.

### 2.4 Affinity of E7 Peptide to Periodontal Cells

GECs, GFs, PDLSCs, and BMSCs were seeded onto the NFG-MS or E7-conjugated NGF-MS (E7-NFG-MS) in Costar Flat Bottom Ultra-Low Attachment 96-well plates at a density of 5 × 10^4^ cells/well, separately. After cultivation for 3 h, the microspheres were transferred to 40 μm cell strainers, gently washed with PBS three times to remove the unadhered cells, and then transferred to a new well in 96-well plates. The cell adhesion rates of each cell type were detected using a cell counting kit-8 (CCK-8, Sigma-Aldrich). Briefly, 100 μL of α-MEM and 10 μL of CCK-8 solution were added into each well and incubated at 37 °C for 1 h. Cell viability was examined by measuring the absorbance at 450 nm using a microplate reader. The cell adhesion rate was calculated by dividing the number of cells adhering on microspheres by the total number of cells seeded in each well. Three samples were included in each group, and the experiment was repeated three times.

### 2.5 Cell Spreading and Morphology Observation

To investigate the effect of the E7 peptide on the morphology of the periodontal cells, the GECs, GFs, PDLSCs, and BMSCs were seeded onto the NFG-MS or E7-NFG-MS in Costar Flat Bottom Ultra-Low Attachment 96-well plates at a density of 1 × 10^4^ cells/well, separately. The microspheres were transferred to a new well in a 24-well plate after cultivation for 1 h to avoid cell aggregation. After cultivation for 3, 6, and 12 h, immunofluorescent staining of the actin cytoskeleton was performed to observe the stretch and morphology of cells on the microspheres. The cell-microsphere constructs were fixed with 4% paraformaldehyde (PFA) at 4 °C for 30 min and permeabilized in 0.3% Triton X-100 for 15 min. After blocking with 20% goat serum and 3% BSA for 1 h, the samples were stained with CF633 Phalloidin (Biotium, USA) at room temperature for 2 h and counterstained with Hoechst (Invitrogen). A confocal laser scan microscope (TCS SP5, Leica, USA) was used to acquire images of the single cell on a microsphere. The cell spreading area was calculated by using Imaris 9.0 and Image-Pro plus 6.0 software. Three samples were collected at each time point, and the experiment was repeated three times.

### 2.6 Competitive Cell Adhesion Assay

GECs, GFs, PDLSCs, and BMSCs were seeded in 6-well plates at a density of 2 × 10^5^ cells/well, separately. Once the cells reached 80–90% confluency, they were prestained for 30 min with cell tracker dye Violet BMQC, Green CMFDA, Orange CMRA, and Deep Red (Thermo Fisher Scientific), as per the manufacturer’s instructions. The cells were washed with PBS, collected by trypsinization, counted, and diluted to a density of 2 × 10^5^ cells/mL. The same quantity of each cell type was evenly mixed, and 200 μL of the mixed cells was seeded onto the NFG-MS or E7-NFG-MS in 24-well plates. After cultivation for 3 h, the nonadhered cells were removed using cell strainers, and the adhered cells were collected by trypsinization, fixed with 4% PFA, and resuspended in PBS for quantitative analysis of the proportion of each type of cells by a flow cytometry assay. Three samples were performed at each time point, and the experiment was repeated three times.

### 2.7 Establishment of a Rat Mandible Periodontal Fenestration Defect Model

The animal research procedure was conducted with the approval of the University Committee on Use and Care of Animals of Texas A&M University College of Dentistry (TAMU-COD). A total of 24 male SD rats aged 8 weeks were randomly assigned to four groups: Empty (*n* = 6), NFG-MS (*n* = 6), Mem + NFG-MS (*n* = 6), and E7-NFG-MS (*n* = 6). The Empty group served as a natural healing control. The NFG-MS group was used to evaluate the effect of microspheres on periodontal regeneration. The Mem + NFG-MS group was the combination of a GTR collagen membrane (Geistlich Bio-Gide) with NFG-MS and was used to compare the effectiveness of E7-NFG-MS for periodontal regeneration. The E7-NFG-MS group was the experimental group.

A rat mandible periodontal defect model was established based on our previously reported periodontal fenestration model with modification.^22^ After anesthesia, the surgical site was prepared by removing the surrounding fur and disinfecting the skin. The surgery was performed under a 10× magnifying stereoscope. An incision (1.5 cm) was made along the mandible inferior border. The buccal gingival tissue was dissected to expose the first and second molars. A no. 2 round bur was used to remove the buccal root of the first molar, followed by a no. 1/2 bur to complete the osteotomy, creating a standard defect size of 3 mm (height) × 2 mm (width) × 1 mm (depth). The microspheres were then carefully implanted into the defect area. The masseter muscle and the skin over the surgical site were sutured in place. After surgery, the rats received analgesia for 3 days and soft diets for 7 days. The rats were sacrificed 8 weeks after surgery, and the samples were harvested for histology, immunohistochemistry, and μ-CT assay.

### 2.8 Histology and Immunohistochemistry Assay

The rat mandibles were fixed in 4% PFA at 4 °C for 48 h, washed with PBS for 1 h, and demineralized in 15% ethylenediaminetetraacetic acid (EDTA) for 6 weeks at room temperature. After ethanol gradient dehydration and paraffin embedding, consecutive coronal sections (4 μm) of the distal root of the first molar were dewaxed and rehydrated for histology and immunohistochemistry analysis. Masson staining was conducted for histologic observation using the Trichrome Stain Kit (Sigma-Aldrich) according to the manufacturer’s instructions. Sirius Scarlet staining was performed to identify the arrangement of the periodontal collagen fibers. Immunohistochemical analysis was performed with a Vectastain Elite ABS-HRP Kit (Vector Laboratories, CA) according to the manufacturer’s instructions. Antibodies included pan-Cytokeratin Antibody (AE1/AE3) (1:100, sc-81714, Santa Cruz Biotechnology) and fibroblast marker (ER-TR7) (1:100, sc-73355, Santa Cruz Biotechnology). The height of the newly formed alveolar bone was measured based on the horizontal line drawn from the lowest point of the PDL of the tooth root. The height of attachment loss was taken as the distance between the cementoenamel junction (CEJ) and the lowest point of the gingival epithelium attached to the root surface. The fibrous tissue height was the distance between the apex of the new alveolar bone and the highest point of the fibrous tissue in the area corresponding to the vertical orientation of the alveolar bone. The functional PDL height was defined as the distance between the lowest point of the PDL and the highest PDL attachment point of the cementum-PDL-alveolar bone functional structure. Histomorphometric analysis was conducted with Image-Pro plus 6.0 software. At least four slides from each sample were used for the measurements.

It is important to note that enamel and the CEJ are imperceptible in the demineralized sections. In rodent histology, the junctional epithelium is typically found in close proximity to or directly at the CEJ.^32,33^ Furthermore, we observed that the crowns of rat teeth exhibit a distinct inward angle at the bottom (toward the tooth crown) of the JE, with the apex of the angle aligning precisely with the bottom of the JE (Figure S1). To ensure consistent results, this study designated the bottom of the JE as the location of the CEJ. Consequently, during the surgical removal of soft tissue from the root surface, we deliberately preserved only a minimal amount of tissue from the bottom of the JE, which was directed toward the tooth crown. The precise location of the apical segment of the junctional epithelium, and by extension, the CEJ, was subsequently determined by evaluating the scant remaining JE tissue and the apex of the internal angle within the tooth crown.

### 2.9 μ-CT Assay

μ-CT scanning of the fixed rat mandible was conducted at a resolution of 10 μm using a μ-CT35 imaging system (Scanco Medical). The scanning data were reconstructed and segmented using Mimics software 21.0 (Materialise, Belgium). The newly formed bone volume was analyzed. The bone density of new bone is relatively lower than that of the original bone. In addition, the connection between the new bone and the original bone tissue is irregular or nonsmooth. Therefore, the ROI of the new bone volume was determined by the boundaries of the new bone tissue through grayscale values and junction morphology on the μCT. The root exposure length was defined as the vertical distance between the CEJ and the lowest point of the exposed root.

### 2.10 Statistical Analysis

Statistical analysis was performed using SPSS software version 16.0 (SPSS, Chicago, IL). The results are presented as mean ± standard deviation. The differences between the four groups were compared using one-way ANOVA with a significance level of *p* < 0.05.

## 3 Results

### 3.1 Synthesis and Characterization of E7-NFG-MS

NFG-MS was fabricated by combining a water-in-oil emulsification process with a thermally induced phase separation technique (Figure 1C). An EDC/NHS chemical cross-linking step was incorporated to enhance the stability of the nanofibrous architecture of the NFG-MS. In our experimental setup, a stirring speed of 800 rpm produced the NFG-MS with diameters ranging between 20 and 150 mm, and the NFG-MS with diameters ranging between 75 and 90 mm were selected for this study. Sulfo-SMCC, a heterobifunctional cross-linker, was employed to conjugate the E7 peptide onto the NFG-MS. The cross-section confocal images of E7-NFG-MS in Figure 1D showed that the E7 peptide, with an average thickness of 6.4 ± 1.2 μm, was evenly conjugated onto the outer layer of the E7-NFG-MS.

**Figure 2:**
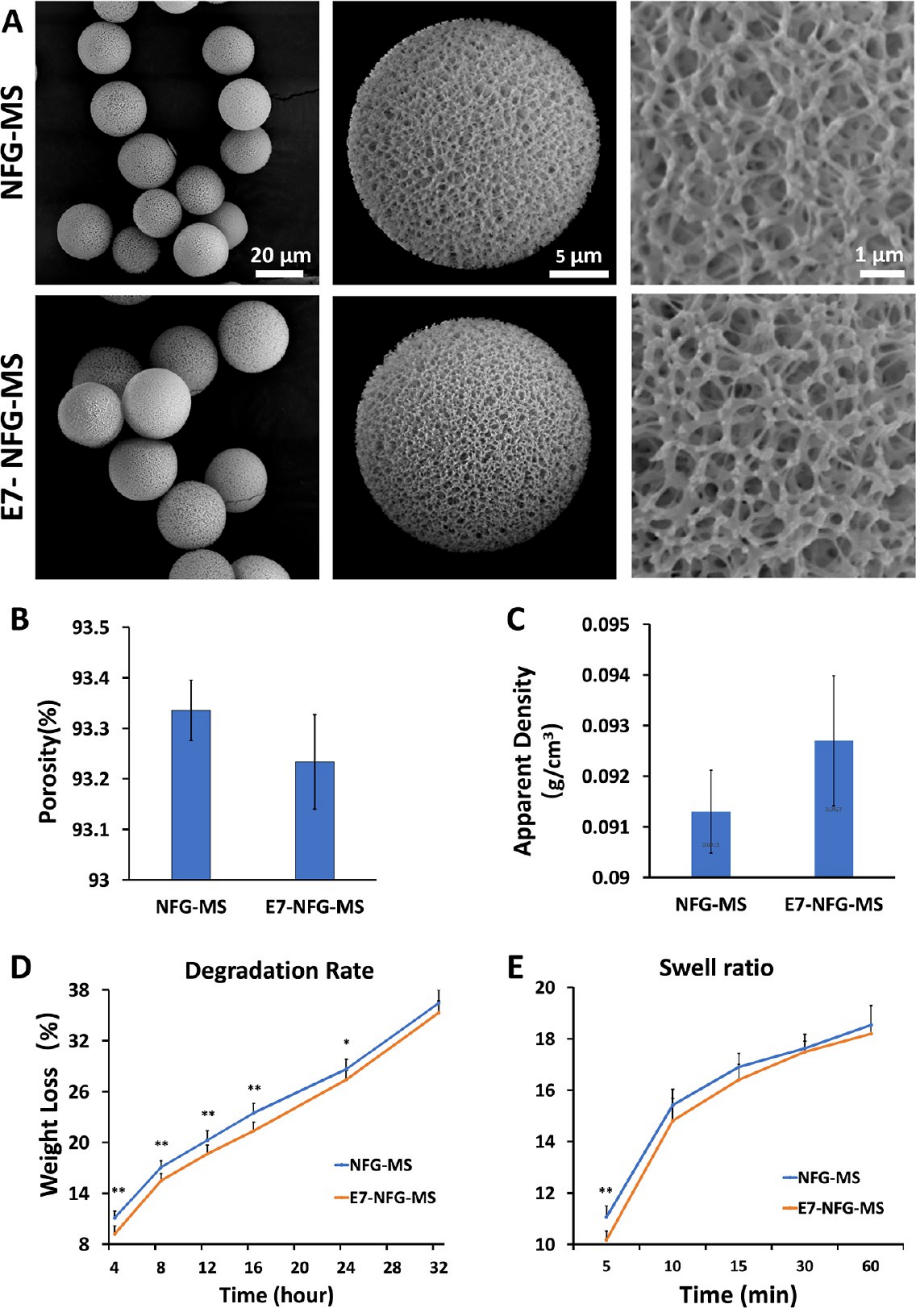
Characterizations of NFG-MS and E7-NFG-MS. (A) SEM images of NFG-MS and E7-NFG-MS. (B–E) Porosity, apparent density, degradation rate, and swell ratio of NFG-MS and E7-NFG-MS. (*P < 0.05, **P < 0.01).

Similar to the NFG-MS, the E7-NFG-MS consisted entirely of nanofibers with an average diameter of 230 ± 42 nm (Figure 2A), which is in the same range as that of natural collagen fibers in bone ECM. The E7-NFG-MS had a high porosity of 93.2 ± 0.1% and a low apparent density of 0.092 ± 0.001 g/cm^3^, which provides sufficient space for ECM deposition and nutrient exchange and minimizes the generation of degradation byproducts when the microspheres are used as injectable scaffolds (Figure 2B,C). The degradation rate of E7-NFG-MS was slightly slower than that of NFG-MS, possibly owing to the E7 peptide covering the enzymatic degradation sites on the surfaces of the microspheres (Figure 2D). The E7-NFG-MS was hydrophilic and had a high equilibrium swelling ratio (Figure 2E). Overall, the conjugation of E7 onto the NFG-MS did not impact the architecture, porosity, or apparent density of the NFG-MS.

### 3.2 Adhesion and Spreading of Periodontal Cells on E7-NFG-MS

**Figure 3:**
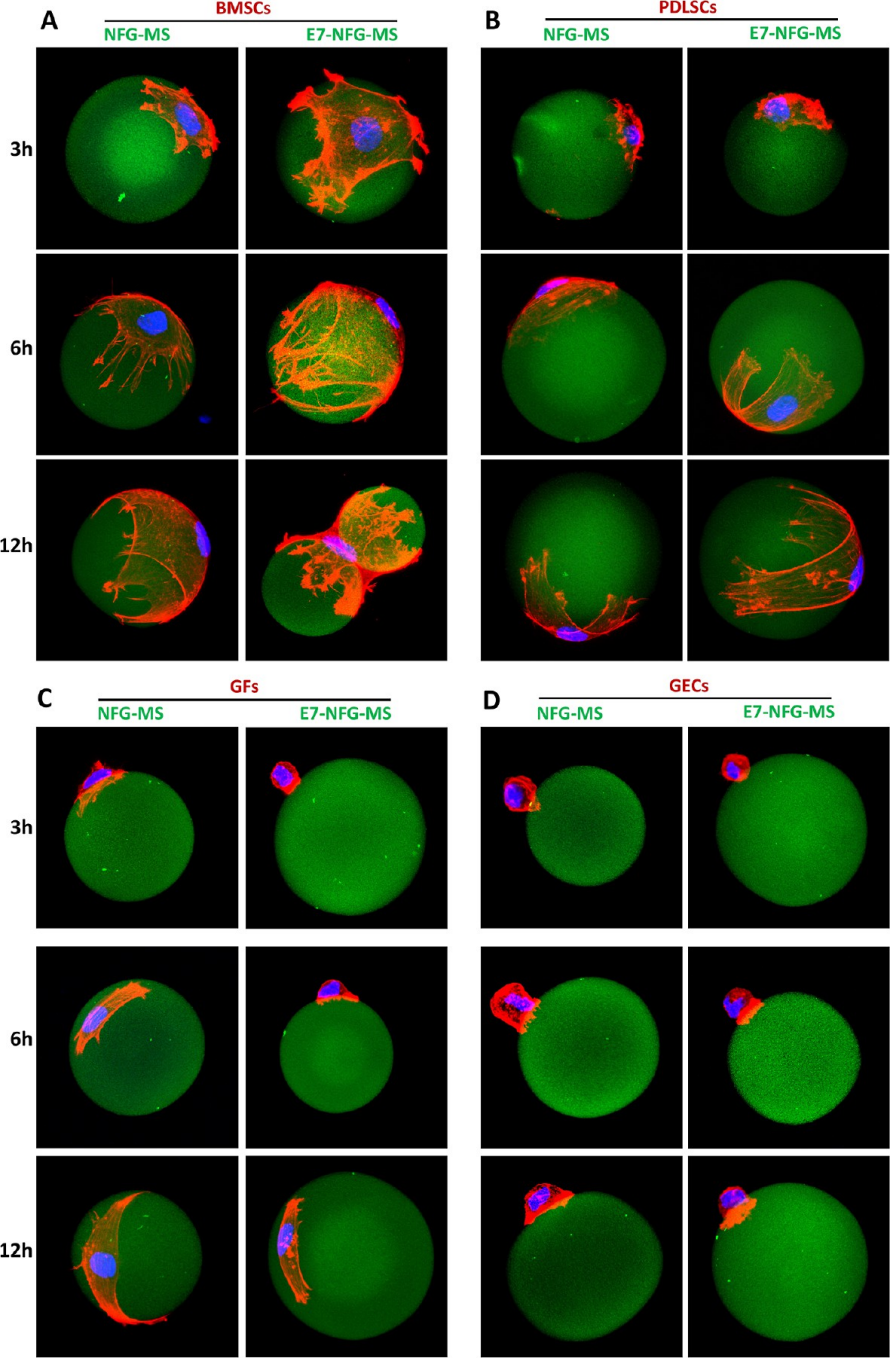
Confocal images of the adhesion and spreading of (A) BMSCs, (B) PDLSCs, (C) GFs, and (D) GECs on NFG-MS and E7-NFG-MS at 3, 6, and 12 h.

To examine the effect of the E7 peptide on cell adhesion, BMSCs, PDLSCs, GFs, and GECs were seeded on the NFG-MS and E7-NFG-MS for 3, 6, and 12 h. The cytoskeleton of each single cell on the microsphere was stained with phalloidine and visualized by confocal laser scanning microscopy. As shown in Figure 3, the morphologies of the 4 cell types on the microspheres were distinctly different. At the same time point, the spreading area of the BMSCs on E7-NFG-MS was significantly greater than that on NFG-MS (Figure 3A). At 6 h, the BMSCs were fully spread, and the cytoskeleton was well-arranged, covering the E7-NFG-MS surface like a turban. At 12 h, the cytoskeleton of a single BMSC spread to connect two adjacent microspheres, indicating the strong and stable adhesion of the BMSC on the E7-NFG-MS. Conversely, the spreading rate of BMSCs on the NFG-MS was substantially slower, and the spreading area of BMSCs on the NFG-MS at 6 h was smaller than that on the E7-NFG-MS at 3 h. While the BMSC on the NFG-MS was fully spread at 12 h, it could not connect two microspheres, as shown in the E7-NFG-MS group. Similarly, PDLSCs on E7-NFG-MS fully spread in 6 h and exhibited aligned cytoskeletons resembling a ribbon around the microspheres, while the PDLSC on NFG-MS remained in a contractive morphology with unclear cytoskeletons (Figure 3B).

Compared to the BMSCs and PDLSCs, the GFs spread much more slowly on E7-NFG-MS (Figure 3C). At 3 and 6 h, the GFs showed a rounded morphology on the E7-NFG-MS with very small spreading areas. At 12 h, the spreading area of the GFs was still small on the E7-NFG-MS, while the GFs displayed an expanding spindle-shape on the NFG-MS. The GECs retained a shrunken, rounded morphology on both the NFG-MS and E7-NFG-MS at 3, 6, and 12 h, suggesting that the nanofibrous architecture of the microspheres inhibited GEC adhesion (Figure 3D). To better observe cell morphology, the individual cell extension images in Figure 3 without microspheres are shown in Figure S2 of Supporting Information.

Quantitative analysis indicated that the addition of E7 onto NFG-MS significantly increased the adhesion of BMSCs and PDLSCs. Specifically, the adhesion rates of BMSCs and PDLSCs at 3 h after cell seeding increased by 65 and 7%, respectively (Figure S3). In addition, the adhesion rates of GFs and GECs decreased by 27 and 47%, respectively. At 12 h, the average spreading area of BMSCs on the E7-NFG-MS increased to 6726 ± 380 μm^2^, while the average spreading areas for the GFs and GECs on the E7-NFG-MS were only 649 ± 48 and 318 ± 67 μm^2^, respectively (Figure S3). These results indicated that E7-NFG-MS selectively promoted the adhesion and spreading of BMSCs and PDLSCs and inhibited the adhesion and spreading of GFs and GECs.

### 3.3 Competitive Adhesion of Periodontal Cells on E7-NFG-MS

**Figure 4:**
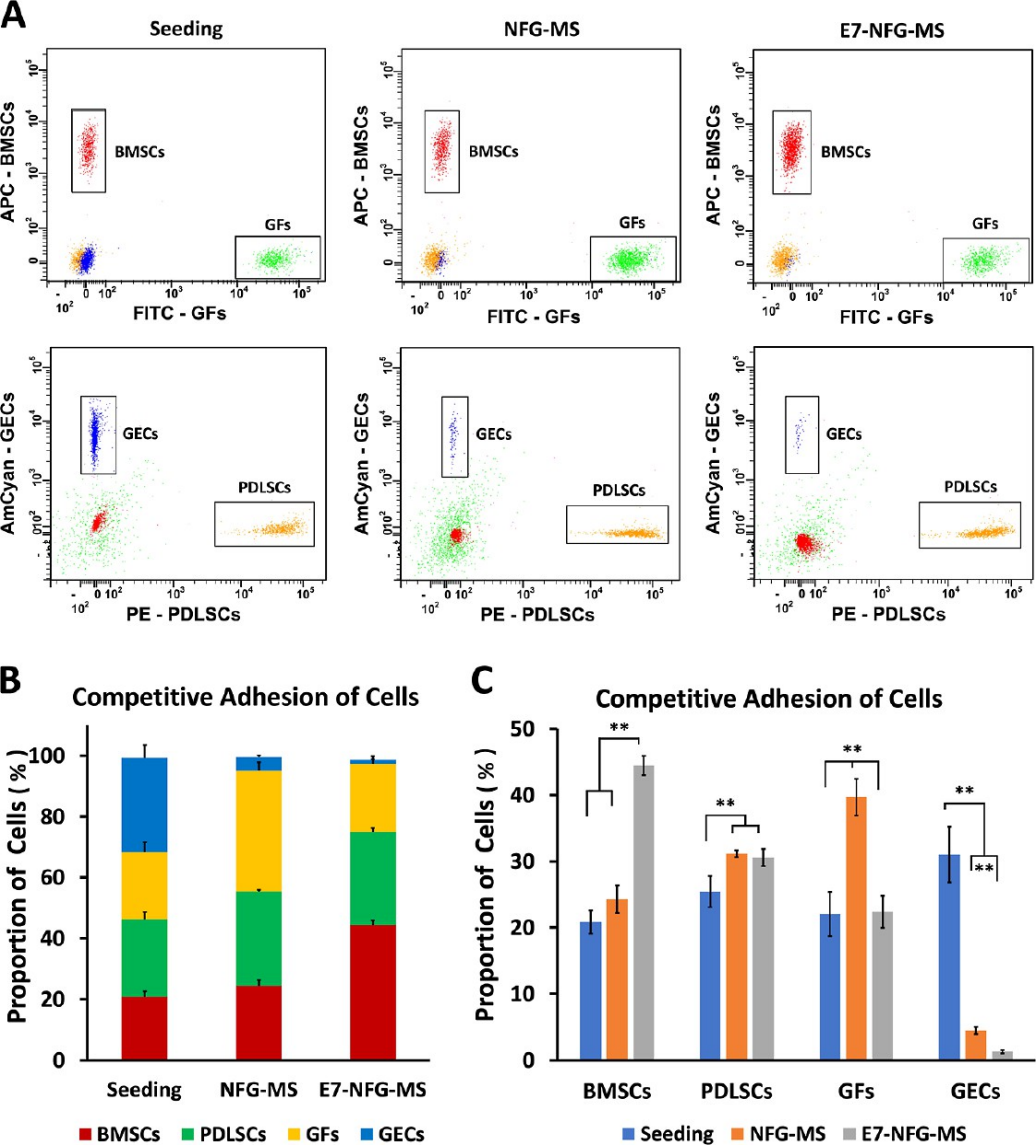
Competitive adhesion of BMSCs, PDLSCs, GFs, and GECs on the NFG-MS and E7-NFG-MS. (A) Flow cytometry scatter plots showing the density of BMSCs, PDLSCs, GFs, and GECs. (B,C) Quantitative analysis of the proportion of BMSCs, PDLSCs, GFs, and GECs. (*P < 0.05, **P < 0.01).

BMSCs, PDLSCs, GFs, and GECs were mixed and seeded onto the microspheres to investigate the competitive adhesion of the cells onto the E7-NFG-MS. Each cell type with the same cell number was labeled with cell-tracking dyes prior to seeding onto the NFG-MS and E7-NFG-MS. At 3 h, the adherent cells were harvested for flow cytometry to analyze the ratio of each type of cell on the microspheres (Figure 4). In the NFG-MS group, the BMSCs and PDLSCs slightly increased the ratios in the total adherent cells, while the number of GFs was significantly increased (Figure 4B). Consequently, the GECs dramatically decreased the proportion to approximately 4.5%. In the E7-NFG-MS group, the competitive adhesion advantage of the GFs was eliminated and competitive adhesion of BMSCs was observed. Specifically, the BMSCs increased by 113.5% compared to the initial seeding numbers. Moreover, the BMSCs in the E7-NFG-MS group were 44.5%, which was 1.8-fold higher than that in the NFG-MS group. The number of PDLSCs in the E7-NFG-MS group was higher than that in the NFG-MS group but was not significant. The number of GFs in the E7-NFG-MS group decreased by 43.6% compared to that in the NFG-MS group. For the GECs, the adhesion ratio was reduced to 1.3% on E7-NFG-MS (Figure 4C). These results further confirmed that E7-NFG-MS specifically enhances the competitive adhesion of BMSCs and suppresses the competitive adhesion of GFs and GECs.

### 3.4 Characterization of the Modified Rat Mandibular Periodontal Defect Model

**Figure 5:**
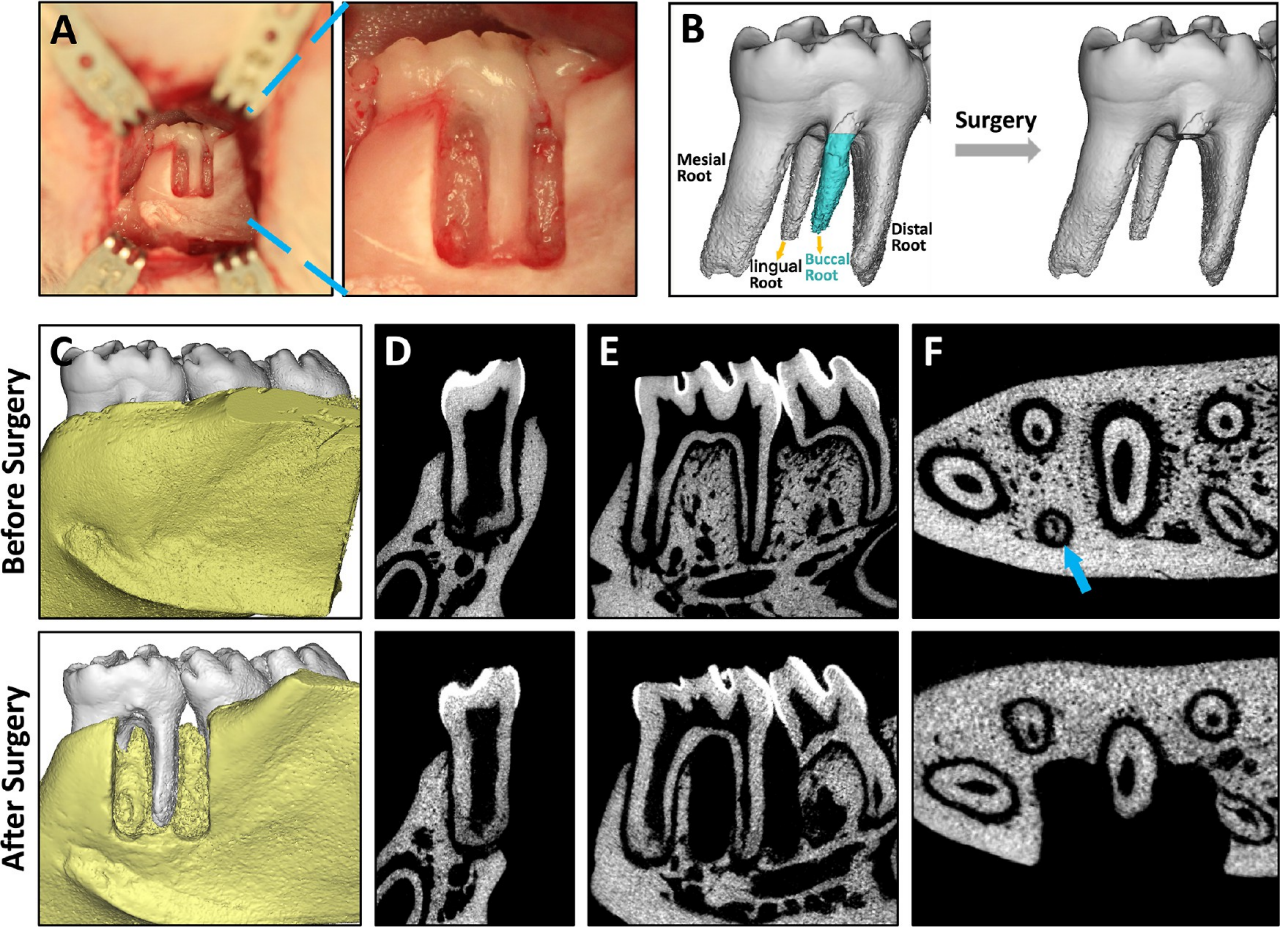
Characterizations of the modified rat mandibular periodontal defect model. (A) Photographs of the rat mandibular periodontal defect model. (B) μ-CT reconstructed images of rat mandibular first molar with the buccal root removed in the defect model. (C) μ-CT reconstructed images of the rat mandible before and after the establishment of the periodontal defect model. (D–F) Gray images of the rat mandible in the coronary, sagittal, and horizontal planes before and after the establishment of the periodontal defect model.

To simulate clinical periodontal defects, a modified rat periodontal fenestration model that connects with the oral cavity was established (Figure 5). The defect had a standard size of 3 × 2 mm (height) × 1 mm (depth), and the PDL on the exposed distal root was scraped off. Corresponding to the clinical classification of periodontal defects,^34^ this modified periodontal defect model is a combination of horizontal bone defect and two-wall defect, with horizontal bone loss of the buccal alveolar bone and remaining mesial and distal alveolar bone wall. As the amount of bone adjacent to the exposed root surface is less than that of the two-wall defect and greater than that of the horizontal bone defect, the prognosis of this periodontal defect model is expected to be worse than that of the two-wall defect and better than that of the horizontal bone defect, both of which remain challenges for clinical treatment. This model was utilized to evaluate the effect of E7-NFG-MS on periodontal regeneration.

### 3.5 μ-CT Analysis of the Effect of E7-NFG-MS on Periodontal Regeneration

**Figure 6:**
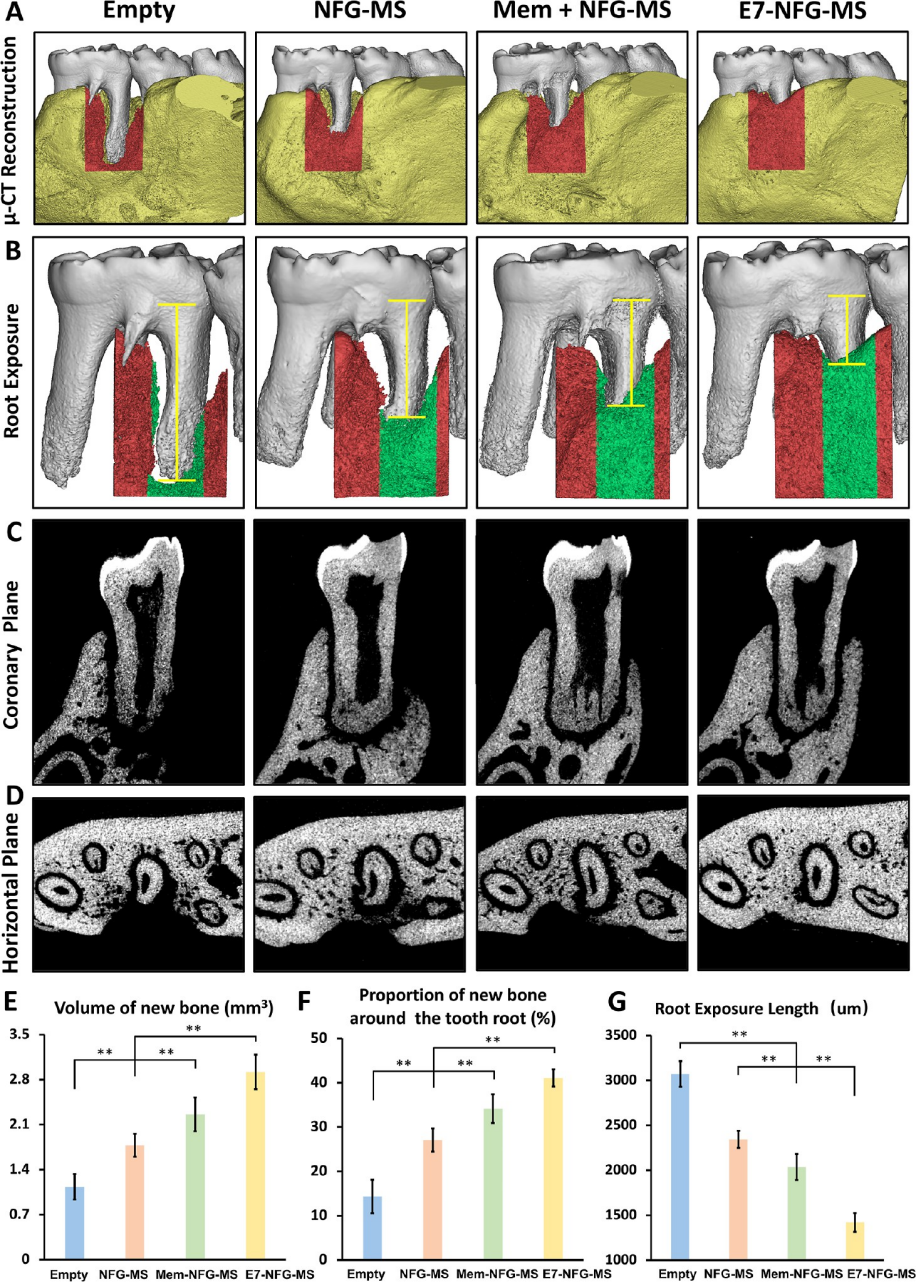
μ-CT analysis of newly formed alveolar bone and tooth root exposure at 8 weeks after surgery. (A) μ-CT reconstructed images of the rat mandible and newly formed bone; the newborn bone is marked in red. (B) μ-CT reconstructed images of the rat mandible first molar and newly formed bone around the root; the newborn bone in the 1 mm zone surrounding the tooth root is marked in green; yellow spacing markers indicate the root exposure length. (C,D) Gray images of the coronary and horizontal planes of the distal root of the first molar. (E–G) Quantitative analysis of new bone volume, the proportion of new bone around the tooth root, and root exposure length. (*P < 0.05, **P < 0.01).

The animals were sacrificed 8 weeks after the surgery, and the samples were harvested for μ-CT assay. The newborn bone in the defect area was segmented and marked in red, as shown in the μ-CT reconstructed images (Figure 6). The E7-NFG-MS group exhibited the greatest amount of total new bone volume, followed by the Mem + NFG-MS, NFG-MS, and Empty groups. The newly formed bone in the E7-NFG-MS, Mem + NFG-MS, and NFG-MS groups was 2.6, 2.0, and 1.6 times higher than that in the Empty group, separately (Figure 6E). Grayscale images in the horizontal and coronary plane displayed that although the defect area was covered with newly formed bone, the newly formed bone on the root surface was limited, with the amount of newly formed bone decreasing as the distance from the root apex increased (Figure 6C,D). Root exposure was greatest on the buccal surface of the root and was farthest from the remaining alveolar bone wall, implying that the migration of stem cells to this area requires a greater distance and time. The proportion of new bone around the tooth root was only 14.3% in the Empty group (Figure 6F). Compared to the Empty control, the proportion of new bone to the roots in the NFG-MS, Mem + NFG-MS, and E7-NFG-MS groups increased to 27.1, 34.2, and 41.1%, respectively.

Enhanced new bone volume surrounding the tooth root is inversely correlated with root exposure (Figure 6B,G). The root of the Empty group was almost entirely exposed in the defect area, with minimal coverage of new bone (Figure 6B). The root exposure length in the NFG-MS and Mem-NFG-MS groups was 2343 ± 95 and 2036 ± 144 μm, respectively. By contrast, the root exposure length in the E7-NFG-MS group decreased to 1420 ± 105 μm, indicating that the E7-NFG-MS effectively promoted new bone formation and reduced the root exposure length in the periodontal defect.

### 3.6 Histological Analysis of the Effect of E7-NFG-MS on Periodontal Regeneration

**Figure 7:**
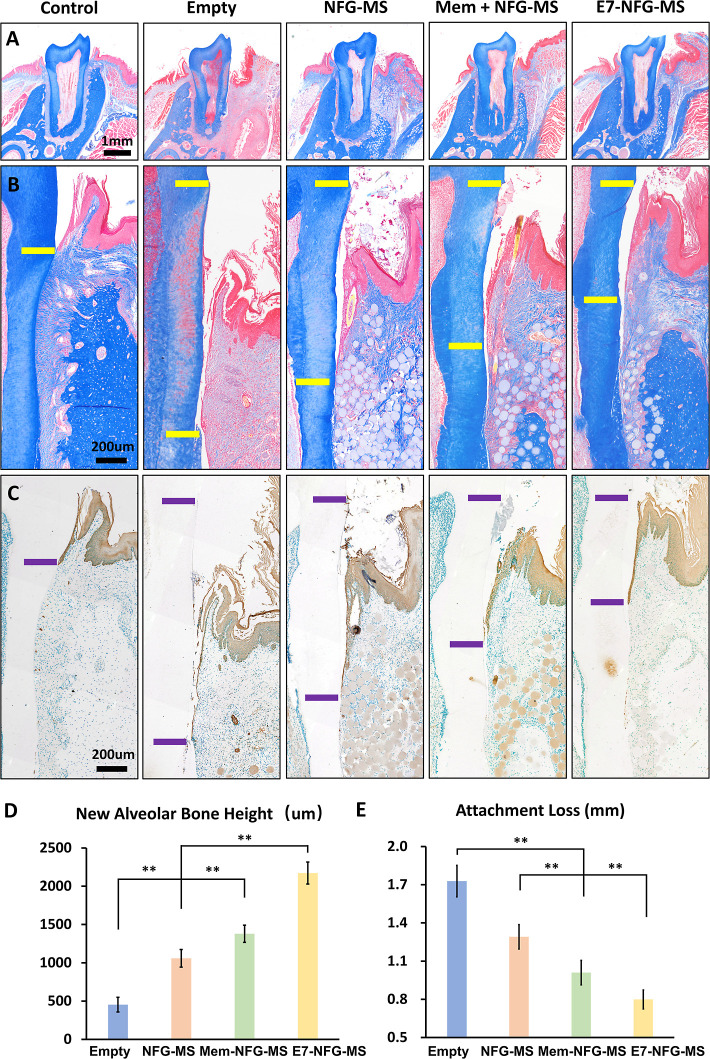
New alveolar formation and attachment loss in the periodontal defect area at 8 weeks after surgery. (A) Masson staining showing the restoration of the periodontal defect area. (B,C) Masson staining and IHC staining for pan-Cytokeratin antibody (AE1/AE3) showing epithelial attachment and attachment loss. Yellow and purple horizontal lines indicate the extent of attachment loss. (D,E) Quantitative analysis of attachment loss and new alveolar height. (*P < 0.05, **P < 0.01).

As shown in Figure 7, Masson’s trichrome staining shows the regenerated tissues, including collagen and bone (blue), keratin epithelial and muscle fibers (red), and cytoplasm (pink). The restored alveolar bone height in the E7-NFG-MS group was significantly higher than that in the Mem + NFG-MS, NFG-MS, and Empty groups (Figure 7A). Specifically, the newly regenerated alveolar bone height in the E7-NFG-MS, Mem-NFG-MS, NFG-MS, and Empty groups was 2172 ± 144, 1378 ± 112, 1059 ± 115, and 453 ± 96 μm, respectively (Figure 7D). Immunohistochemistry staining targeting the epithelial marker “AE1/AE3” was conducted to assess the extent of attachment loss. As shown in Figure 7B,C, the E7-NFG-MS group exhibited the least attachment loss, whereas the Empty group had the most attachment loss. The quantitative results showed that the attachment loss in the E7-NFG-MS, Mem-NFG-MS, NFG-MS, and Empty groups was 799 ± 75, 1010 ± 96, 1291 ± 94, and 1728 ± 124 μm, respectively (Figure 7E).

**Figure 8:**
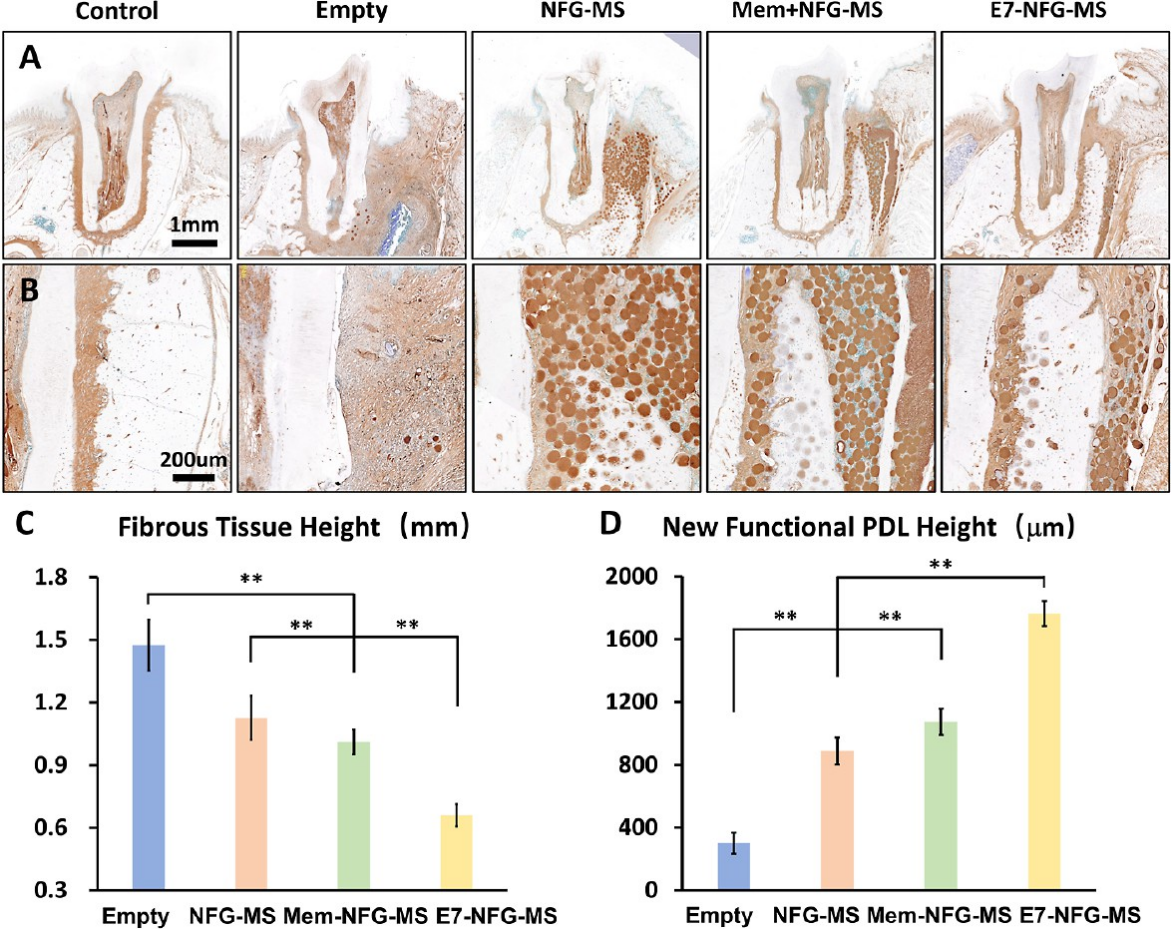
Fibrous tissue formation in the periodontal defect area at 8 weeks after surgery. (A,B) IHC staining for fibroblast marker (ER-TR7) showing the formation and distribution of fibrous tissue in the periodontal defect area. (C,D) Quantitative analysis of fibrous tissue height and new functional PDLC height. (*P < 0.05, **P < 0.01).

Immunohistochemistry for the fibrous tissue marker “ER-TR7” revealed that the positive area of fibrous tissue in the healthy control group predominantly overlapped with the PDL, with a minor proportion of fibrous tissue located in the gingival connective tissue (Figure 8A,B). In contrast, the defect area in the Empty group exhibited complete filling with fibrous tissue. In the NFG-MS, Mem + NFG-MS, and E7-NFG-MS groups, the fibrous tissue was primarily situated around and above the newly formed alveolar bone. The fibrous tissue height in the Empty control, NFG-MS, Mem + NFG-MS, and E7-NFG-MS groups was 1475 ± 122, 1127 ± 105, 1012 ± 59, and 660 ± 53 μm, respectively (Figure 8C). Additionally, there was an inverse relationship between the regenerated fibrous tissue height and the functional PDL height. Specifically, the functional PDL height in the Empty control, NFG-MS, Mem + NFG-MS, and E7-NFG-MS groups was 301 ± 67, 888 ± 85, 1074 ± 83, and 1764 ± 80 μm, respectively (Figure 8D).

**Figure 9:**
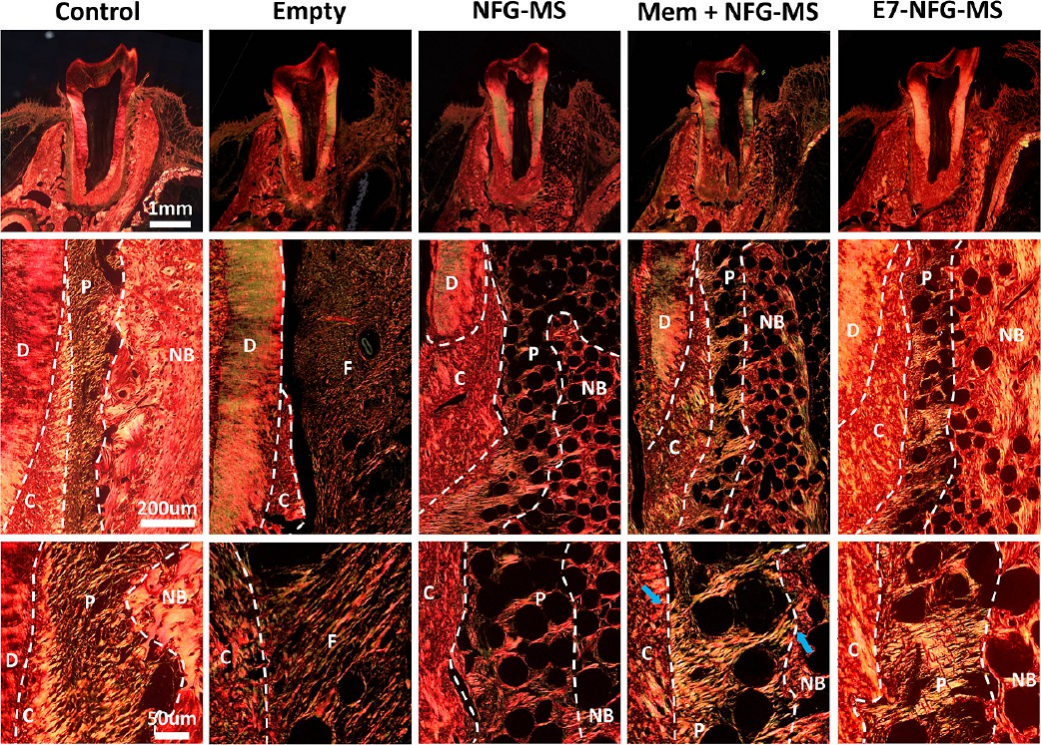
Functional periodontal tissue formation in the periodontal defect area at 8 weeks after surgery. Sirius Scarlet staining showing the distribution and arrangement of collagen fibers in the periodontal defect area. D, dentin; C, cementum; P, periodontal ligament; F, fibrous tissue; NB, newly formed bone.

Sirius Scarlet staining was utilized to evaluate the arrangement of collagen and the formation of the functional structure of periodontals (Figure 9). In the healthy control group, a well-arranged PDL connecting the tooth root to the alveolar bone through Sharpey’s fibers was observed. The PDL was embedded in the cementum and alveolar bone, enabling supportive function. In the Empty group, the defect area had little PDL structure formation and displayed only disordered collagen fibers. The PDL structure arranged in an orderly manner was regenerated in the NFG-MS, Mem + NFG-MS, and E7-NFG-MS groups. The regenerated PDL anchored to the newly formed root cementum and alveolar bone via Sharpey’s fibers, suggesting the formation of functional periodontal tissues. Notably, more functional periodontal structures were formed in the E7-NFG-MS group compared to the NFG-MS and Mem + NFG-MS groups, as evidenced by the increased height of the newly formed functional PDL (Figure 8D). Furthermore, the collagen bundles within the newly formed cementum, PDL, and alveolar bone in the E7-NFG-MS group exhibited denser packing and greater maturity, manifested by their orange-red coloration.^35^ Altogether, these results show that the E7-NFG-MS effectively promoted the restoration of alveolar bone height, reduced PDL loss and fibrous tissue formation, and ultimately facilitated the formation of functional periodontal structures.

## 4 Discussion

Periodontitis is the most prevalent oral disease and is characterized by the destruction of periodontal supporting tissues.^1^ Although GTR is the most commonly used technique for periodontal tissue regeneration in clinical practice, it can only effectively treat narrow intrabony and class II mandibular furcation defects and is unpredictable for other types of periodontal defects. Moreover, complications associated with GTR surgery are frequent and negatively affect clinical outcomes.^36^ In this work, we propose a novel selective cell affinity approach that modulates competitive cell adhesion to accelerate periodontal regeneration in a clinically challenging periodontal defect. We designed and fabricated bioinspired microspheres that are conjugated with E7 peptide that prefers the adhesion of BMSCs. The E7-conjugated bioinspired microspheres (E7-NFG-MS) selectively promoted the adhesion of BMSCs and PDLSCs while inhibiting the adhesion of GFs and GECs. In a modified rat periodontal defect model, we demonstrated the superiority of E7-NFG-MS over the conventional GTR technique in facilitating periodontal regeneration.

Functional periodontal regeneration is challenging, partially owing to its intricate anatomy.^7^ Injectable scaffolds can easily fill any irregularly shaped defects and therefore are appealing for periodontal tissue regeneration.^5^ The selection of nanofibrous gelatin microspheres as the injectable scaffold for periodontal regeneration in this work is based on their distinctive advantages.^22^ First, the NFG-MS are composed entirely of gelatin nanofibers, simulating the chemical composition and physical architecture of collagen, the main component of periodontal ECM. Second, the NFG-MS possess low apparent density and high porosity, therefore, providing sufficient space for cell growth and ECM deposition.^37^ Moreover, the low apparent density of NFG-MS results in minimal degradation products after the microspheres are degraded. Third, the NFG-MS exhibit excellent biocompatibility and osteoconductivity.^24,38^ Last, but not least, the macropores between the NFG-MS facilitate cell migration, proliferation, and vascular inward growth, while the micropores between the nanofibers in the NFG-MS promote ECM deposition.^39^ Therefore, we selected NFG-MS as the substrate and conjugated the surface with the E7 peptide to create a favorable microenvironment for periodontal regeneration.

There are four primary sources of cells involved in periodontal regeneration: GECs, GFs, PDLSCs, and BMSCs. The outcome of regeneration relies on the growth conditions of these four cell types.^8−10^ In a spontaneous healing process, GECs migrate fast and are the first to attach to the root surface, forming a long junctional epithelium that hinders other cells from attaching to the root surface and thus impeding periodontal regeneration. GFs migrate at a rate second only to that of GECs and form fibrous tissues after contacting the root surface. Followed by the migrations of GECs and GFs, PDLSCs occupy the root surface and differentiate into cementoblasts, PDL cells, and osteoblasts and form new periodontal tissues. BMSCs are the last cell type migrating into the defect area, and they differentiate into osteoblasts to form new alveolar bone. Based on the spontaneous periodontal healing principle, conventional GTR technology utilizes a physical barrier membrane to prevent the apical migration of GECs and GFs while providing a secluded space for the inward migration of PDLSCs and BMSCs. However, the GTR technology using nonabsorbable and absorbable barrier membranes has various limitations.^12^ The innovation of this study is to replace the physical GTR membrane barrier with a biological barrier for functional periodontal tissue regeneration.

To achieve this goal, we incorporated the E7 peptide onto the surfaces of nanofibrous microspheres for selective cell adhesion. The E7 peptide is a novel peptide with the amino acid sequence of “EPLQLKM” identified through phage display technology, which has a high specific affinity to BMSCs.^18^ The E7 peptide has recently been applied to specifically enhance the adhesion and proliferation of BMSCs.^19,40−42^ In addition, the E7-modified collagen was found to selectively capture BMSCs over fibroblasts and immune cells.^20^ Those reports suggest that the E7 peptide is likely to function as a biological barrier to selectively enrich BMSCs while excluding epithelia and fibroblasts. In this study, we conjugated the E7 peptide onto the surface of NFG-MS using a surface chemical coupling process, which has the advantage of long-term stability of E7 on the NFG-MS surface over physical adsorption. The in vitro data indicated that E7-NFG-MS significantly facilitated the adhesion of the BMSCs and PDLSCs while preventing the adhesion of the GECs and GFs. In particular, the E7-NFG-MS substantially increased the adhesion rate of BMSCs to 44.5% while dramatically decreasing the adhesion rate of GECs to 1.3% in the competitive adhesion assay of the four cell types (Figure 4). Additionally, the cell spreading experiments showed that BMSCs achieved complete spreading on E7-NFG-MS within only 6 h, whereas the GECs remained as crumpled spheres up to 12 h with limited adhesion areas on both NFG-MS and E7-NFG-MS (Figure 3). These in vitro data demonstrated that the E7-NFG-MS selectively promoted the adhesion and spreading of BMSCs and PDLSCs but inhibited the adhesion of GECs and GFs.

Cells adhere to ECM/scaffolds through binding of cell adhesion proteins to specific ligands on the ECM/scaffolds. The interactions between cell adhesion proteins and ligands determine cell adhesion and thus the affinity of the ECM/scaffold for the cells. BMSCs, PDLSCs, and GFs reside in a microenvironment of the ECM that is mainly composed of collagen and fibronectin (FN) fibrils that contain the Arg-Gly-Asp (RGD) sequence, a common cell recognition motif for integrins.^43^ Gelatin (a hydrolyzed derivative of collagen) contains RGD motifs in its molecular chains, which is attributed to the high adhesion of GFs in the NFG-MS group. GECs adhere to ECM/scaffolds primarily through hemidesmosomes (HDs), similar to basal ECs adherence to the basement membrane.^32^ The basement membrane, a soft ECM beneath epithelial cells, comprises predominantly laminin and type IV collagen. Laminin serves as the ligand that interacts with integrin α6β4 and is an indispensable component of HD formation. The lack of structural congruence between gelatin and the laminin ligand could elucidate the lowest GEC adhesion in the NFG-MS group. The E7 peptide was identified through an affinity selection technique called phage display biopanning that screens peptides binding to BMSCs in a positive screening process and excludes peptides binding to fibroblasts in a negative screening process.^18^ Furthermore, the incorporation of the E7 peptide obscures the presentation of the innate RGD domain on the surface of the E7-NFG-MS, which means fewer binding sites for GFs and GECs and thus reduced competitive adhesion compared to BMSCs.

The rat fenestration model has been widely employed in periodontal regeneration experiments.^44^ However, the standard fenestration defect model is isolated from the oral environment, excluding negative variables such as gingival tissue ingrowth and bacterial contamination of the wound, which is completely different from the clinical periodontal defect induced by periodontitis. In this study, we modified the fenestration model to better mimic the clinical situation by connecting the defect to the oral cavity. The spontaneous healing of this modified periodontal defect model is characterized as a long junctional epithelium combined with substantial connective tissue repair and limited alveolar bone, PDL, and cementum formation (Empty group, Figures 7–9). Clearly, the defect of this model is more challenging for functional periodontal regeneration than the narrow intrabony defect and the class II mandibular furcation defect. We developed a challenging fenestration model to evaluate the effect of E7-NFG-MS on periodontal regeneration. The in vivo experiment demonstrated that the E7-NFG-MS served as an excellent biological barrier to prevent the inward growth of GECs and GFs, as well as a conducive scaffold to promote the inward growth of BMSCs and PDLSCs. Eight weeks after surgery, the E7-NFG-MS group significantly improved functional periodontal regeneration, including enhanced new bone formation, reduced root exposure, and diminished attachment loss. Notably, the quality and quantity of the regenerated periodontal tissues in the E7-NFG-MS group were remarkably better than those in the group involved with the conventional GTR technique (Mem + NFG-MS group).

In the Mem+NFG-MS group, a bioabsorbable collagen membrane was combined with NFG-MS to treat the periodontal defect. Similar to the problems that were reported for the clinical application of the GTR techniques,^17,36^ we encountered two difficulties when using the Mem + NFG-MS for periodontal regeneration. First, the collagen membrane easily collapsed in the defect area and hindered the growth of BMSCs and PDLSs. Second, we investigated the problem of collagen membrane exposure after surgery. The prevalence of membrane exposure is a major clinical complication associated with GTR, occurring in 50 to 100% of cases.^45,46^ In our study, primary flap closure failure was the main cause of membrane exposure, resulting in bacterial invasion and wound infection. There was a high incidence of gingival tears during suturing as well as gingival recession and flap displacement induced by mandible movement after surgery. To ensure an objective assessment of the effect of GTR on periodontal regeneration, we excluded the samples with the incidence of complications in this study. It is noteworthy that the incidence of complications was not observed in the E7-NFG-MS group, indicating the promise of E7-NFG-MS as a new GTR material for periodontal regeneration.

Several reasons could contribute to the better outcome of the E7-NFG-MS. First, the microspheres were in close contact with the tooth root and could hinder the migration of GECs along the root surface and reduce the possibility of GFs migrating inward. This is supported by the result that the attachment loss and fibrous tissue height in the NFG-MS group were smaller than those in the Empty group. However, NFG-MS could not selectively inhibit the adhesion of GFs and GECs on their surfaces. The integration of the E7 peptide on the surface of NFG-MS inhibited the adhesion and migration of GECs and GFs, leading to further reduced attachment loss and fibrous tissue height in the E7-NFG-MS group. Significantly more functional periodontal structure was formed in the E7-NFG-MS group than in the Mem + NFG-MS group, which is possibly because the E7-NFG-MS (a biological barrier) provided a better nutrient exchange environment during tissue regeneration than the Mem + NFG-MS (a physical barrier). In addition, a recent study indicated that E7 peptide interacted with BMSCs and increased the expressions of SDF-1 and CXCR4, which further promoted the homing of BMSCs.^42^ Together, the enrichment of BMSCs in the periodontal defect resulted in abundant new bone regeneration in the E7-NFG-MS group.

It should be noted that although the NFG-MS and E7-NFG-MS had similar degradation rates in vitro, much fewer E7-NGF-MS residues were observed in vivo compared to the NFG-MS and Mem + NFG-MS (Figures 7–9). Our previous study demonstrated that the local microenvironment played a pivotal role in scaffolding degradation. For example, when the scaffolds were implanted in the periodontal defects of diabetic and healthy rats, separately, more scaffolding residues and less new bone formation were observed in the diabetic group than in the healthy group.^21^ In this study, the selective affinity of E7-NGF-MS scaffolds for BMSCs created a favorable microenvironment for periodontal regeneration, which, in turn, affected the scaffold degradation.

## 5 Conclusions

We developed a novel BMSC specific affinity biomaterial by conjugating the E7 peptide with injectable nanofibrous microspheres. The bioinspired microspheres selectively enhanced the adhesion of BMSCs and repelled the adhesion of GFs and GECs, functioning as a biological barrier. By reversing the cell attachment sequence, E7-NFG-MS facilitated BMSCs to occupy the periodontal defect, leading to significant improvements in periodontal regeneration. The in vivo experiment using a modified rat periodontal defect model confirmed the effectiveness of E7-NFG-MS in enhancing functional periodontal regeneration. The E7-NFG-MS represents a promising approach for periodontal regeneration.

## Data Availability

The data that support the findings of this study are available from the corresponding author upon reasonable request.

## Supporting Information Available

Masson staining for CEJ location; confocal images of four types of cells on microspheres at 3, 6, and 12 h; and adhesion rate and spreading area of four types of cells on microspheres at 3, 6, and 12 h (PDF)
