# Supplementary material for: E7-Conjugated Bio-Inspired Microspheres as a Biological Barrier for Guided Tissue Regeneration
Source: ACS Appl Mater Interfaces. 2023 Dec 8;15(50):58136–50. doi: 10.1021/acsami.3c12213 (PMC10862379; doi:10.1021/acsami.3c12213)
Supplement: Supplementary file 1 — am3c12213_si_001.pdf [file am3c12213_si_001.pdf]

Supporting Information

E7-Conjugated Bio-Inspired Microspheres as a Biological Barrier for Guided Tissue Regeneration

Zhiai Hu[1], Xin Rong[1], and Xiaohua Liu[1, 2*]

Department of Biomedical Sciences, Texas A&M University School of Dentistry, Dallas, TX 75246

Chemical and Biomedical Engineering Department, University of Missouri, Columbia, MO 65211

*Correspondence to:

Xiaohua Liu, PhD

Professor

Department of Chemical and Biomedical Engineering

Roy Blunt NextGen Precision Health Building

1030 Hitt Street, Columbia MO 65211

Phone: 573-882-6497

Email: xlz2y@missouri.edu

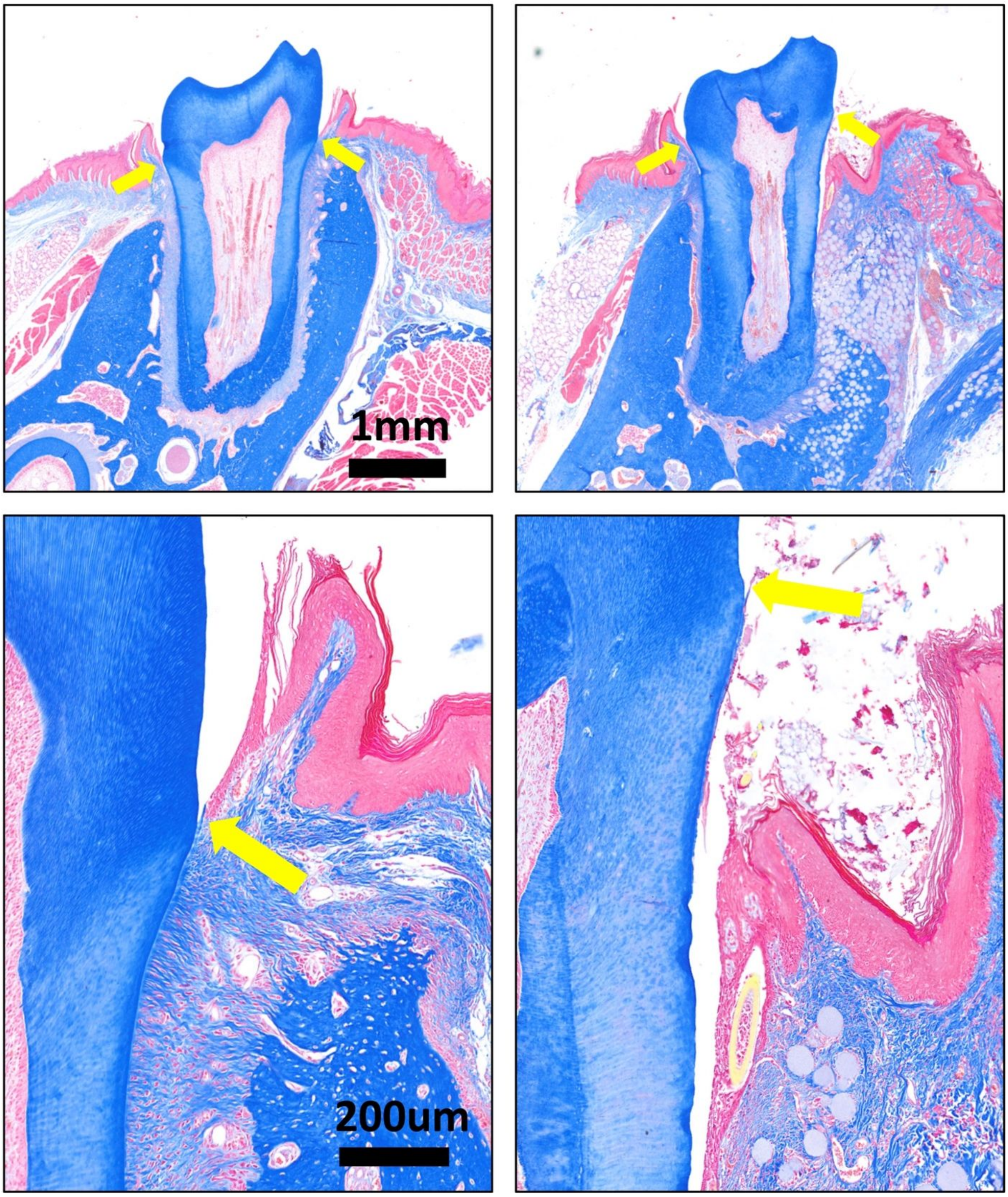

Figure S1. Masson staining images show the location of the cementoenamel junction (CEJ): the crowns of rat teeth exhibit a distinct inward angle at the bottom (towards the tooth crown) of the junctional epithelium (JE), with the apex of this angle aligning precisely with the bottom of the JE. The yellow arrows point to the location of CEJ.

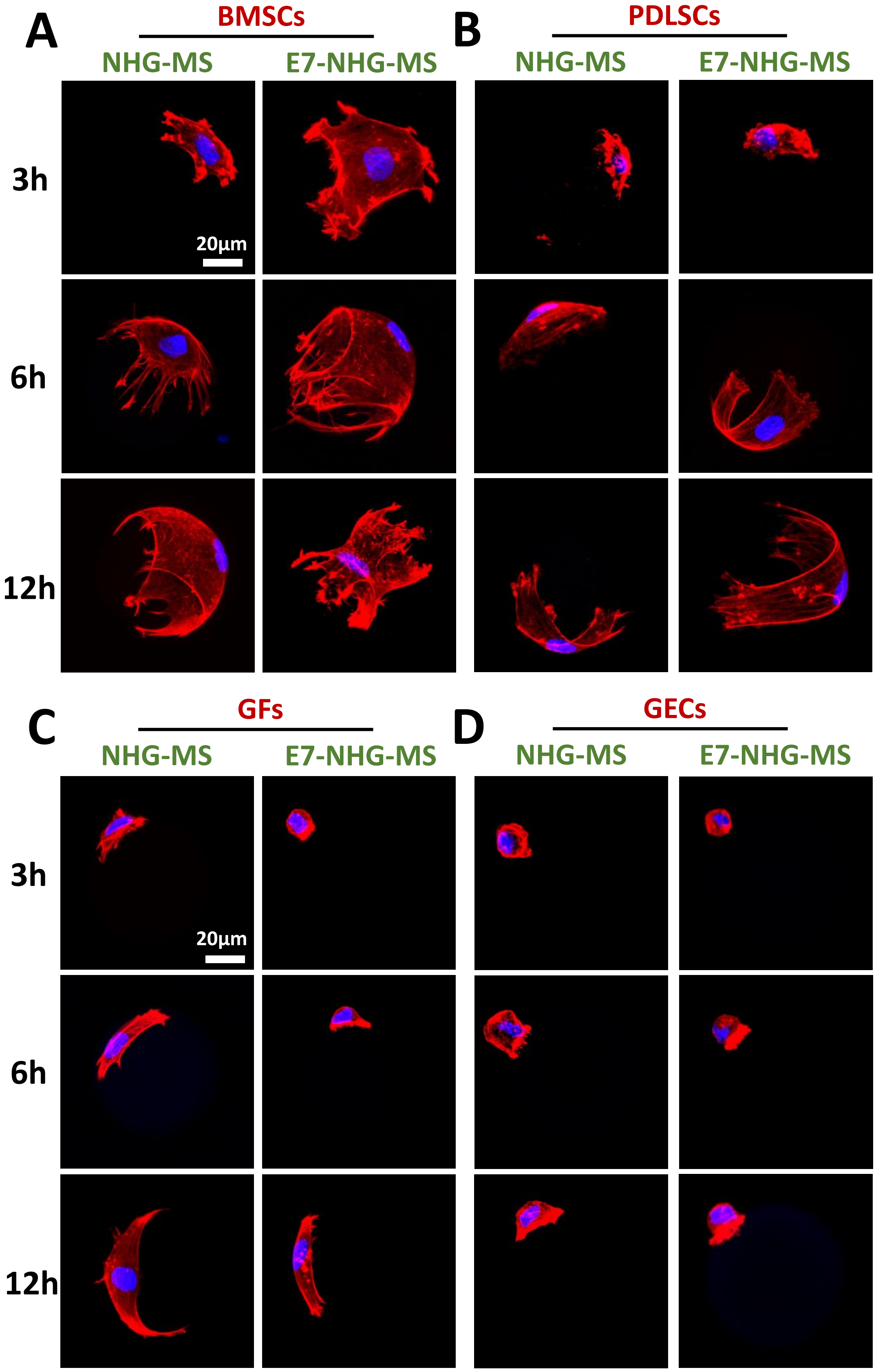

Figure S2. Confocal images of (A) BMSCs, (B) PDLSCs, (C) GFs, and (D) GECs seeded on NHF-MS and E7-NFG-MS at 3, 6, and 12h. The combined cell-sphere images are shown in Figure 3 of the manuscript.

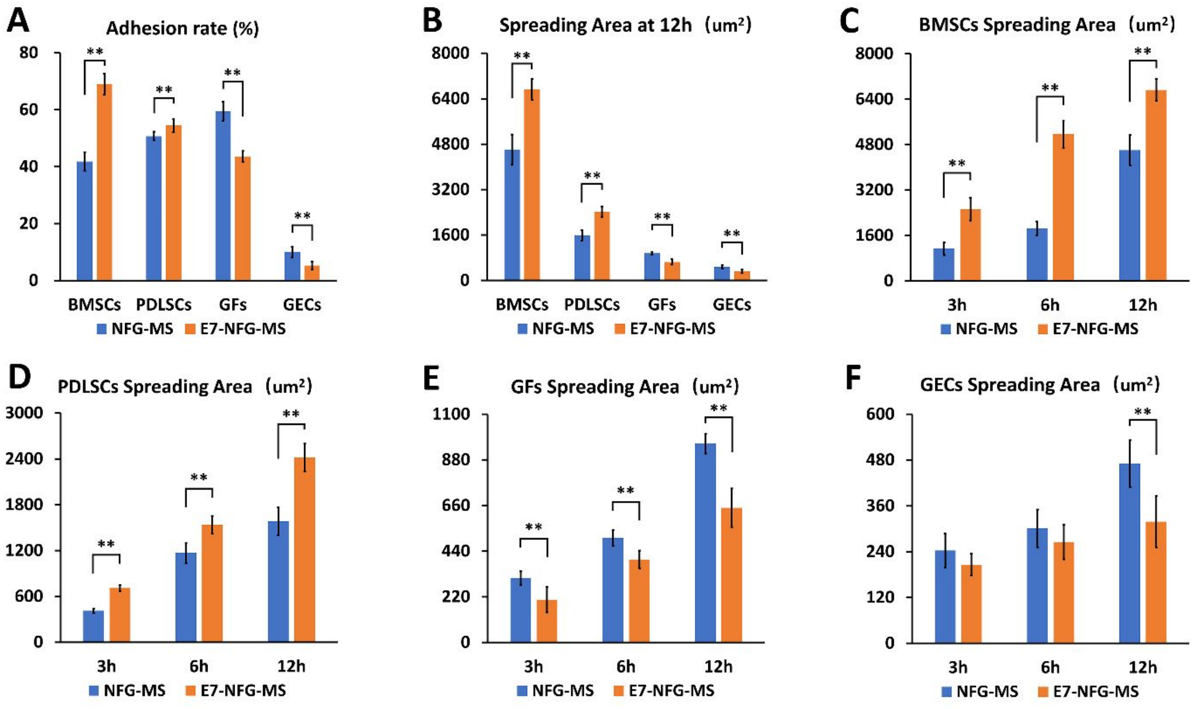

Figure S3. (A) Adhesion rate of BMSCs, PDLSCs, GFs, and GECs on NFG-MS and E7-NFG-MS at 3h, respectively. (B-F) Spreading area of BMSCs, PDLSCs, GFs, and GECs on NFG-MS and E7-NFG-MS at 3, 6 and 12h.
